# The Internet-Based Conversational Engagement Clinical Trial (I-CONECT) in Socially Isolated Adults 75+ Years Old: Randomized Controlled Trial Protocol and COVID-19 Related Study Modifications

**DOI:** 10.3389/fdgth.2021.714813

**Published:** 2021-08-25

**Authors:** Kexin Yu, Katherine Wild, Kathleen Potempa, Benjamin M. Hampstead, Peter A. Lichtenberg, Laura M. Struble, Patrick Pruitt, Elena L. Alfaro, Jacob Lindsley, Mattie MacDonald, Jeffrey A. Kaye, Lisa C. Silbert, Hiroko H. Dodge

**Affiliations:** ^1^Suzanne Dworak-Peck School of Social Work, University of Southern California, Los Angeles, CA, United States; ^2^Edward R. Roybal Institute on Aging, University of Southern California, Los Angeles, CA, United States; ^3^Layton Aging and Alzheimer's Disease Center, Department of Neurology, Oregon Health & Science University, Portland, OR, United States; ^4^Department of Systems, Populations and Leadership, University of Michigan School of Nursing, Ann Arbor, MI, United States; ^5^Mental Health Service, Veterans Affairs Medical Center Ann Arbor Healthcare System, Ann Arbor, MI, United States; ^6^Research Program on Cognition and Neuromodulation Based Interventions, Department of Psychiatry, University of Michigan, Ann Arbor, MI, United States; ^7^The Institute of Gerontology, Wayne State University, Detroit, MI, United States; ^8^Department of Health Behavior and Biological Sciences, School of Nursing, University of Michigan, Ann Arbor, MI, United States; ^9^Department of Psychiatry, University of Wisconsin-Madison, Madison, WI, United States; ^10^The School of Psychological Science, Oregon State University, Corvallis, OR, United States; ^11^Syneos Health, Portland, OR, United States

**Keywords:** ADRD, behavioral intervention, randomized controlled trial, trial protocol, cognitive health, social interaction, technology–ICT, social isolation and loneliness

## Abstract

**Background:** Increasing social interactions through communication technologies could offer a cost-effective prevention approach that slows cognitive decline and delays the onset of Alzheimer's disease. This paper describes the protocol of an active project named “Internet-based conversational engagement clinical trial (I-CONECT)” (ClinicalTrials.gov: NCT02871921). The COVID-19 pandemic related protocol modifications are also addressed in the current paper.

**Methods:** I-CONECT is a multi-site, assessor-blind, randomized controlled behavioral intervention trial (RCT). We aim to randomize 320 socially isolated adults 75+ years old [160 Caucasian and 160 African American participants, 50:50 split between those with normal cognition and mild cognitive impairment (MCI)] recruited from the community to either the video chat intervention group or the control group (1:1 allocation). Those in the video chat group receive a computer and Internet service for the duration of the study, which they use to video chat with study staff for 30 min/day 4×/week for 6 months (high dose), and then 2×/week for an additional 6 months (maintenance dose). Both video chat and control groups have a brief (about 10 min) telephone check-in with study staff once per week. The primary outcome is the change in global cognitive function measured by Montreal Cognitive Assessment (MoCA) from baseline to 6 months. Secondary outcomes include changes in cognition in memory and executive function domains, emotional well-being measured by NIH Toolbox emotional battery, and daily functional abilities assessed with the Revised Observed Tasks of Daily Living (OTDL-R). Eligible participants have MRIs at baseline and 6 months. Participants contribute saliva for genetic testing (optional consent), and all video chats, weekly check-in calls and neuropsychological assessment sessions are recorded for speech and language analysis. The pandemic halted research activities and resulted in protocol modifications, including replacing in-person assessment with remote assessment, remote deployment of study equipment, and revised targeted sample size.

**Discussion:** This trial provides user-friendly hardware for the conversational-based intervention that can be easily provided at participants' homes. The trial aspires to use age and culture-specific conversational materials and a related platform developed in this trial for enhancing cognitive reserve and improving cognitive function.

## Introduction

It is well-established that social isolation (small social network and lack of social contact) and loneliness (dissatisfaction with the frequency and quality of social contact) can lead to adverse health outcomes (1, 2), including dementia (3–5). Social isolation has been supported as having a more significant impact on the onset of dementia than some known medical conditions, such as diabetes (6). A recent report by Lancet Commissions showed that 2% of dementia cases could be prevented if we could eliminate social isolation, which is 1% higher than the rate reported for diabetes (6). Although the underlying mechanisms of the association between social connectedness and cognition is not well-established, larger social networks and socially active lifestyles (as opposed to being socially isolated) may enhance cognitive reserve (7), and thereby provide a buffer against cognitive decline associated with aging as well as dementia pathological burden (8–13). Thus, enhancing social connectedness or interactions can be a possible intervention strategy to slow down age-associated cognitive decline as well as to delay the symptomatic expression of ADRD. Since delaying the onset of clinical symptoms of dementia even for a few years can have a large impact on the prevalence of dementia (14), developing sustainable easy-to-start social interaction strategies for those with chronic illness and/or who are home-bound (i.e., those at risk of cognitive decline) is of high public health importance. Randomized controlled trials (RCTs) with clearly specified element(s) and doses of social engagement are needed to clarify the mechanism of the protective function of social engagement, networks and connectedness on cognitive function, and ultimately to translate this knowledge into data-driven actionable intervention programs against cognitive decline. The population effect size of increasing social engagement on delaying dementia disease progression could exceed that of current FDA approved medications for Alzheimer's disease.

To addresses the above need, in our previous National Institute on Aging (NIA) funded pilot randomized controlled behavioral clinical trial, we developed a conversation-based social interaction cognitive stimulation protocol delivered by trained interviewers. In the study, we hypothesized that conversational interactions, i.e., the core component of social interactions, could enhance cognitive resrve, and thereby improve or sustain cognitive functions. Daily 30 min face-to-face communications with trained conversational staff were conducted over a 6-week trial period in the intervention group using personal computers, webcams, and a user-friendly interactive Internet interface, while control participants received only weekly phone contacts (15). A large touch screen monitor with a pop-up window was used to show daily stimuli pictures to spark conversations, allowing even the oldest old participants with no previous experience using PC/mouse/Internet or those with low motivation for participating in behavioral interventions to be easily engaged in conversational interactions. This user-friendly setting also eliminated the potential confounding effect of cognitive stimulation from learning to use a PC. This phase I feasibility study (ClinicalTrials.gov: NCT01571427) demonstrated high adherence in an older adult population (mean age 80 years) and efficacy in improving language-based executive functions among those with normal cognition and mild cognitive impairment (MCI), and psychomotor speed among those with MCI, suggesting that this intervention could be promising for delaying cognitive decline (15). Based on this encouraging result, we are currently conducting a phase II study with a longer intervention period and larger sample size, targeting socially isolated older adults aged 75 and older.

In the ongoing RCT, we target socially isolated and lonely older adults because cognitive function can be enhanced further when activities are novel to individuals (16). Socially isolated older adults are likely to benefit from conversation-based online interventions. Furthermore, loneliness may be mitigated by social interactions and thereby improve cognitive function. Therefore, the benefit of our intervention on cognitive functions is expected to be greatest for those who are socially isolated and lonely with limited opportunities to talk with others.

In this paper, we introduce the protocol of the active project called “Internet-based conversational engagement clinical trial (I-CONECT), www.i-conect.org” (ClinicalTrials.gov: NCT02871921), a multi-site, assessor-blind, randomized controlled behavioral intervention trial. Increasing daily social contact through communication technologies could offer a cost-effective home-based prevention approach that slows cognitive decline and delays the onset of Alzheimer's Disease and Related Dementias (ADRD), thereby reducing the overall societal burden of dementia.

The I-CONECT trial started recruitment and data collection in July 2018. During the second year of study recruitment, the COVID-19 pandemic hit the USA. The duration of trial before the pandemic was about 19 months, from July 2018 to March 2020. The pandemic halted research activities and resulted in protocol modifications, including replacing in-person assessment with remote assessment, remote deployment of study equipment, pausing the MRI assessments, and revising sample size for the main analysis. These COVID-19 pandemic related protocol modifications are also addressed in the current paper.

## Methods

### Objective and Hypotheses

**Figure 1:**
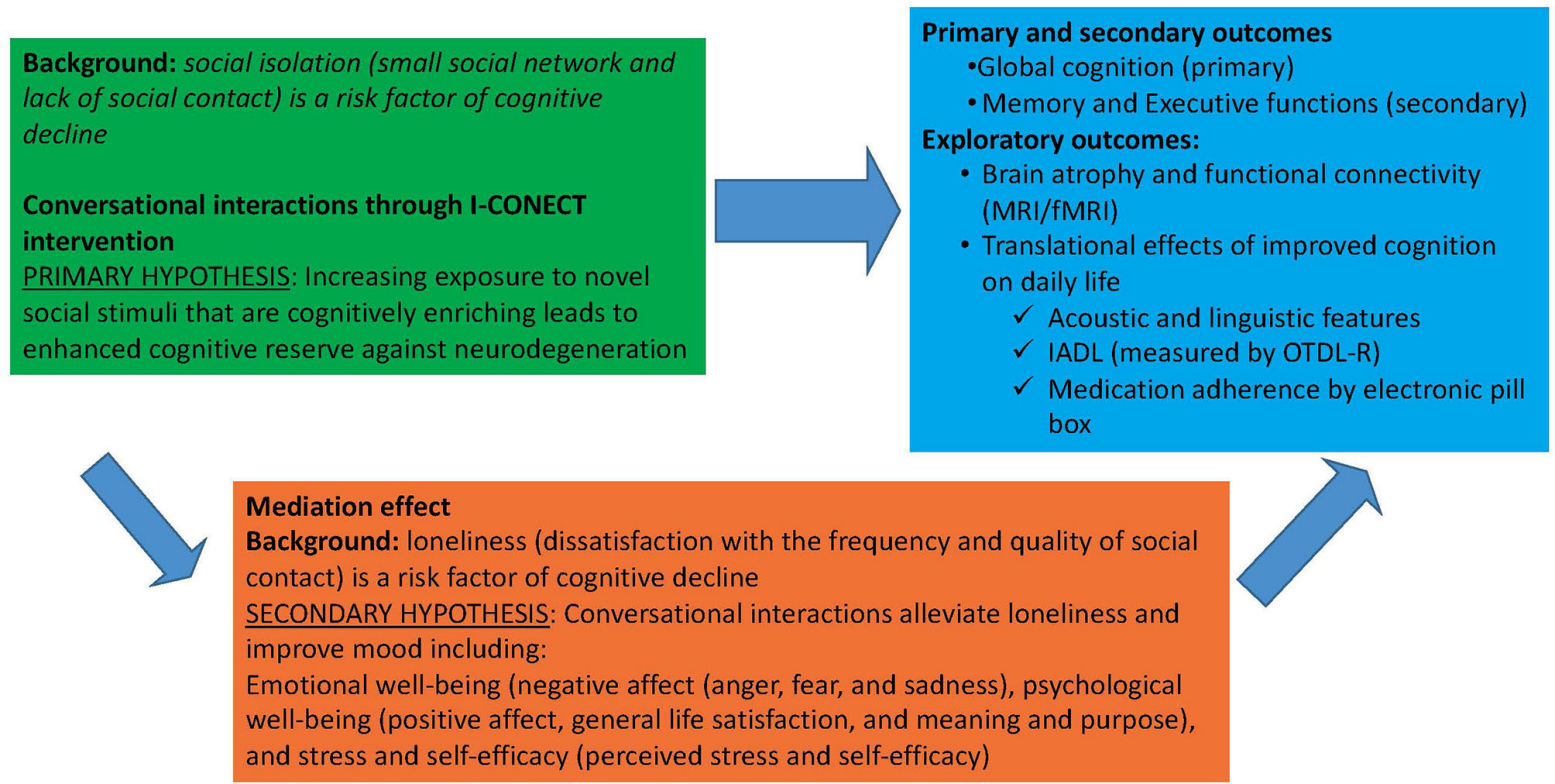
I-CONECT project efficacy assessments: Direct effect of social interaction on cognition and mediating effects through psychological well-being.

The main objective of the I-CONECT trial is to investigate the extent to which online conversational interactions can prevent cognitive decline among socially isolated older adults with normal cognition or MCI. Figure 1 illustrates the direct and indirect pathways of the intervention effects on cognitive outcomes. Our primary hypothesis is that within each cognitive group (normal and MCI) the experimental group will experience less cognitive decline or even improvement in global cognitive function (primary outcome) and executive and memory functions (secondary outcomes) compared to the control group. Our secondary hypothesis is that if this intervention improves psychological well-being, then the intervention efficacy can be partly mediated by such improvements. Translational effects on daily function including improvement in IADL and changes in speech and language characteristics, and changes in brain connectivity measured by fMRI will also be examined under exploratory analyses (discussed in detail later in the Outcomes section).

### Study Design and Participants

The I-CONECT, (ClinicalTrials.gov: NCT02871921) aims to examine whether conversations through user-friendly video chats with multiple trained conversational staff can enhance cognitive functions and emotional well-being. This study is an extension of the previous pilot trial (15, 17). The experimental group receives 30 min video chats with trained conversational staff 4 times/week for the first 6 months (high dose) followed by 2 times/week for an additional 6 months (maintenance dose) and weekly check-in calls that monitor social activities and adverse health events. The control group receives only weekly check-in calls. Participants are recruited from Portland, Oregon (mainly targeting Caucasian participants), and Detroit, Michigan (mainly targeting African American participants) in the United States. A conversation-based behavioral intervention might work differently given various cultural and social backgrounds of the participants. Hence, we aim to diversify the study sample by recruiting African American older adults for half of the study participants so that we can assess the potential difference in intervention efficacy by race. Study approval was obtained from the Institutional Review Board at the Oregon Health & Science University (OHSU) (IRB STUDY00015937) using a single IRB process. OHSU is the home institution of this project. All participants are required to provide written informed consent and are included in the analysis (experimental and control groups).

### Inclusion/Exclusion Criteria

Table 1 lists details of the inclusion and exclusion criteria. Inclusion criteria are age equal to or above 75, normal cognition or mild cognitive impairment (MCI) assessed by the trial neuropsychologist based on cognitive testing at screening, and socially isolated as defined by at least one of the following: (1) score ≤ on the 6-item Lubben Social Network Scale (LSNS-6) (18), (2) engages in conversations lasting 30 minutes or longer no more than twice per week, per subject self-report, (3) answers “often” to at least one question on the 3-item UCLA Loneliness Scale (19) (see Appendix for detail information on measurements used to define being socially isolated and lonely). Exclusion criteria included (1) having dementia, (2) moderate to severe depressive symptoms operationally defined as a 15-item Geriatric Depression Scale (GDS-15) score >7 (20), (3) current alcohol or substance abuse, (4) unstable medical conditions, (5) active systemic cancer within 5 years of the screening visit, or (6) surgery that required full sedation with intubation within 6 months of screening. Further detail is described in ClinicalTrials.gov (# NCT02871921). We aim to recruit 50:50 split between those with MCI and normal cognition.

**Table 1:** Inclusion and exclusion criteria.

**Inclusion criteria**
1) Age 75 or older
2) Consent to participate in the study and to receive MRI (if safely and comfortably able to receive MRI, and if MRI is available at the study site)
3) Socially isolated, defined by at least one of the following:
i. Score ≤12 on the 6-item Lubben Social Network Scale (LSNS-6) (18)
ii. Engages in conversations lasting 30 min or longer no more than twice per week, per subject self-report
iii. Answers “Often” to at least one question on the Hughes et al. Three-Item UCLA Loneliness Scale (19)
4) Adequate vision to use study technology and complete all neuropsychological tests throughout the study, defined by the following:
i. Able to see well enough to read a newspaper, wearing glasses if needed but not using a magnifying glass
5) Adequate hearing to use study technology and complete all neuropsychological tests throughout the study, defined as:
i. Able to hear well enough to complete the telephone screening
6) Sufficient ability to understand English in order to complete protocol-required testing
7) Normal cognition or mild cognitive impairment (MCI), as assessed by the trial neuropsychologist
8) Sufficiently able to comply with protocol assessments and procedures, in the opinion of the investigator
**Exclusion criteria**
1) Identified as having dementia based on either of the following criteria:
i. Self-reported diseases associated with dementia, such as Alzheimer's disease, vascular dementia, Lewy body dementia, frontotemporal dementia, normal pressure hydrocephalus, or Parkinson's disease
ii. Diagnosis of dementia by trial neuropsychologist
2) Anticipating major change in living arrangement within the upcoming year
3) Severely depressed, operationally defined as a 15-item GDS score > 7 (20)
4) Significant disease of the central nervous system, such as brain tumor, seizure disorder, subdural hematoma, or significant stroke, per subject report
5) Current (within 2 years of screening) alcohol or substance abuse
6) Unstable or significantly symptomatic psychiatric disorder, such as major depression, schizophrenia, post-traumatic stress disorder, or bipolar disorder
7) Unstable or significantly symptomatic cardiovascular disease, such as coronary artery disease with frequent angina, or congestive heart failure with shortness of breath at rest
8) Unstable insulin-dependent diabetes mellitus, defined as meeting any of the following criteria:
i. Received a diagnosis of Type 1 Diabetes
ii. Started taking insulin within 3 months of the screening visit
iii. Hospitalization for hypoglycemia within 1 year of screening
9) Active systemic cancer within 5 years of the screening visit (Gleason Grade <3 prostate cancer and non-metastatic skin cancers are acceptable)
10) Surgery that required full sedation with intubation within 6 months of screening (sedation for minor procedures is acceptable)
11) More than one overnight hospital stay within 3 months of the screening visit
12) Any other condition that, in the opinion of the investigator, is severe enough to cause study participation to have a negative impact on participant or study team rights or well-being

We originally targeted those aged 80 and older, but due to a significant challenge in recruiting African American subjects in this age group in Detroit, we lowered the age criterion to 75 after 6 months from the start of the recruitment. Additionally, answering “often” to at least one question on the 3-item UCLA Loneliness Scale was added as a criterion after 10 months from the start of the recruitment to include those who failed to meet the social isolation criteria using LSNS-6 and frequency of conversations, but still expressed isolation (lack of companionship, being left out, or feeling isolated) (19). Social isolation (small social network and lack of social contact) and loneliness (dissatisfaction with the frequency and quality of social contact) capture two different states even though they are often correlated (1, 2). This addition of frequent experience of loneliness as an inclusion criterion is in line with the secondary hypothesis that the impact of our intervention on cognitive functions is partly explained by changes in emotional well-being. This change provides an opportunity to examine which group of elderly can benefit most from our study; socially isolated and lonely, socially isolated only, or lonely without social isolation.

### Recruitment Sources and Approaches

This study recruits via direct outreach to potential subjects (strategies may include telephone calls, direct mail, and in-person distribution of recruitment materials). Potential subjects may be identified for direct outreach from publicly available data (e.g., voting records), word-of-mouth referrals, or through relationships with community partners, including Meals on Wheels People, a non-profit organization that delivers meals to low income or home-bound seniors and Area Agency on Aging (AAA) which provides various services to home-bound seniors. The study also collaborate with the Healthier Black Elders Registry in Detroit, which is maintained by the Michigan Center for Urban African American Aging Research (MCUAAAR) (https://mcuaaar.org/cores/community-liaison-and-recruitment-core/participant-resource-pool/) to facilitate recruitment of African American participants. Study advertisements are also used, including flyers distributed and/or posted in the community, the www.I-CONECT.org study website, write-ups for publication (e.g., in community newsletters), and social media postings and online advertisements (Facebook).

Permission for recording each trial session (audio and video recording) is included in the consent form to participate in the study, but genotype assessment (information on APOE ε4) is made optional to alleviate participant privacy concerns. Six Data and Safety Monitoring Board (DSMB) members were selected before the data who are not in the same institutions as the PIs and Co-investigators of the project and included two members from the funding source (National Institutes of Health).

#### Randomization

Randomization is conducted by a data manager who are not analyze study outcomes. Participants are randomized in a 1:1 ratio to the experimental or control conditions balancing the following factors: age (75–80, 80–85, 85–90, 90+), sex, years of education (high school completion vs. below high school education), and cognitive status (normal vs. MCI) and MoCA score (MoCA score: 23 and above vs. lower than 23). A minimization algorithm is used for randomization (21). This method successfully achieved balancing multiple factors in our previous project (15). APOE ε4 information is not be used as a balancing factor, but if enough APOE ε4 positive subjects are recruited, we will conduct a stratified analysis by APOE ε4 status since the intervention efficacy could differ (22).

#### Clinical Diagnosis of Normal vs. MCI

In the first face-to-face interview, we use the MoCA (the Montreal Cognitive Assessment) to obtain a rough estimate of cognitive status and exclude those with suspected dementia (23, 24). We initially used MoCA <16 as a cutoff score (for the first 3 months of our recruitment), but later we found that quite a few subjects, especially those with lower education, scored lower than 16, yet were functionally independent and had Clinical Dementia Rating Scale (CDR) ≤ 0.5 (25). Therefore, we use MoCA as a rough estimate, and if the subject appears not demented, we complete the screening home visit. If the participants meet other inclusion/exclusion criteria, then a full neuropsychological test battery (listed later) is administered. Upon completion of the neuropsychological test battery (and collection of other necessary information), the test results are evaluated at a consensus meeting among study neurologists and neuropsychologists and clinically determined as MCI or not, based on a standardized approach used in National Institute of Health (NIH)-funded Alzheimer's Disease Centers across the US (26–29). Within 6 weeks of the 1st face-to-face interview, we randomize subjects into control or experiment groups and start sessions until we reach the targeted sample size (i.e., rolling based recruitment).

#### Blindness

The assessors are blind to participants' group assignments. We ensure blindness by having a total separation between assessors and staff who engage in conversational sessions. Assessors are not allowed to discuss any issues with participants that reveal whether the subject is in an experimental or control group. Experienced and trained research associates conduct baseline, post-trial (6 month), and 1 year follow-up assessments.

#### Compensation

All participants who consent to participate receive $50 at screening. Individuals who consent to be screened yet do not meet the inclusion criteria also receive the compensation. Those who receive MRI testing receive $100 for the first MRI visit and $100 for the second MRI visit. Participants receive $50 if they complete testing at the Month 6 time point. Participants receive $75 if they complete testing at the Month 12 time point. The compensations are not pro-rated in the event that a subject discontinues the study early for any reason. In total, a participant who completes the study could receive either $175 (no MRI) or $375 (two MRI visits). These amounts are carefully determined so that the compensation does not constitute undue inducement and also allow participants to retain federal and state income assistance, if applicable.

### Intervention Program

Briefly, the experimental group engages in 30 minutes semi-structured conversations with trained and standardized conversational staff 4 days per week for 6 months (high dose), followed by twice per week for an additional 6 months (maintenance dose). Both intervention and control groups receive a phone call once per week (~10 min) to assess their changes in health and monitor the level of social interaction. This weekly phone call also serves to retain control group participation. Conversational sessions (both audio and video) and weekly calls (audio) are recorded and stored on HIPPA compliant servers at OHSU. We examine the efficacy during the first 6 months (high dose period) and the following 6 months (maintenance dose period) separately. During the maintenance dose period, we aim to examine whether we can maintain gains or improve primary and secondary outcomes beyond those obtained during the high dose period. However, due to the COVID-19 pandemic, the additional follow-ups beyond the first 6 months have been discontinued for the majority of participants (discussed later in detail).

#### Standardization of Interviewer Skills (Conversation Sessions Through PC/Webcam)

In our previous project, we developed a systematic standardization approach which includes (1) practice with other conversational staff and senior volunteers before the actual trial (at least 40 h of training), (2) recording conversational sessions and listening to them for quality control and analyzing word counts generated by interviewer/interviewee. Additionally (3) every Monday, we assess negative and positive affects using International Positive and Negative Affect Schedule Short Form (PANAS) immediately before and after the 30 min conversation and compare the change in scores across interviewers (30, 31). We examine whether a specific interviewer has higher than the average changes in affect items. (4) The proportion of words spoken by interviewers relative to participants is also used as a tool to standardize conversational sessions. Based on our previous pilot project, we aim to keep word counts by the conversational staff at or below 30% of the total word counts to provide ample opportunities for participants to talk. The deviation observed in the number of spoken words by the participant/interviewer during recorded conversations serves as a metric to improve standardization of individual interviewer's interview skills. Each participant has opportunities to talk with different staff each week to retain their attention and motivation.

#### Development of User-Friendly Device

**Figure 2:**
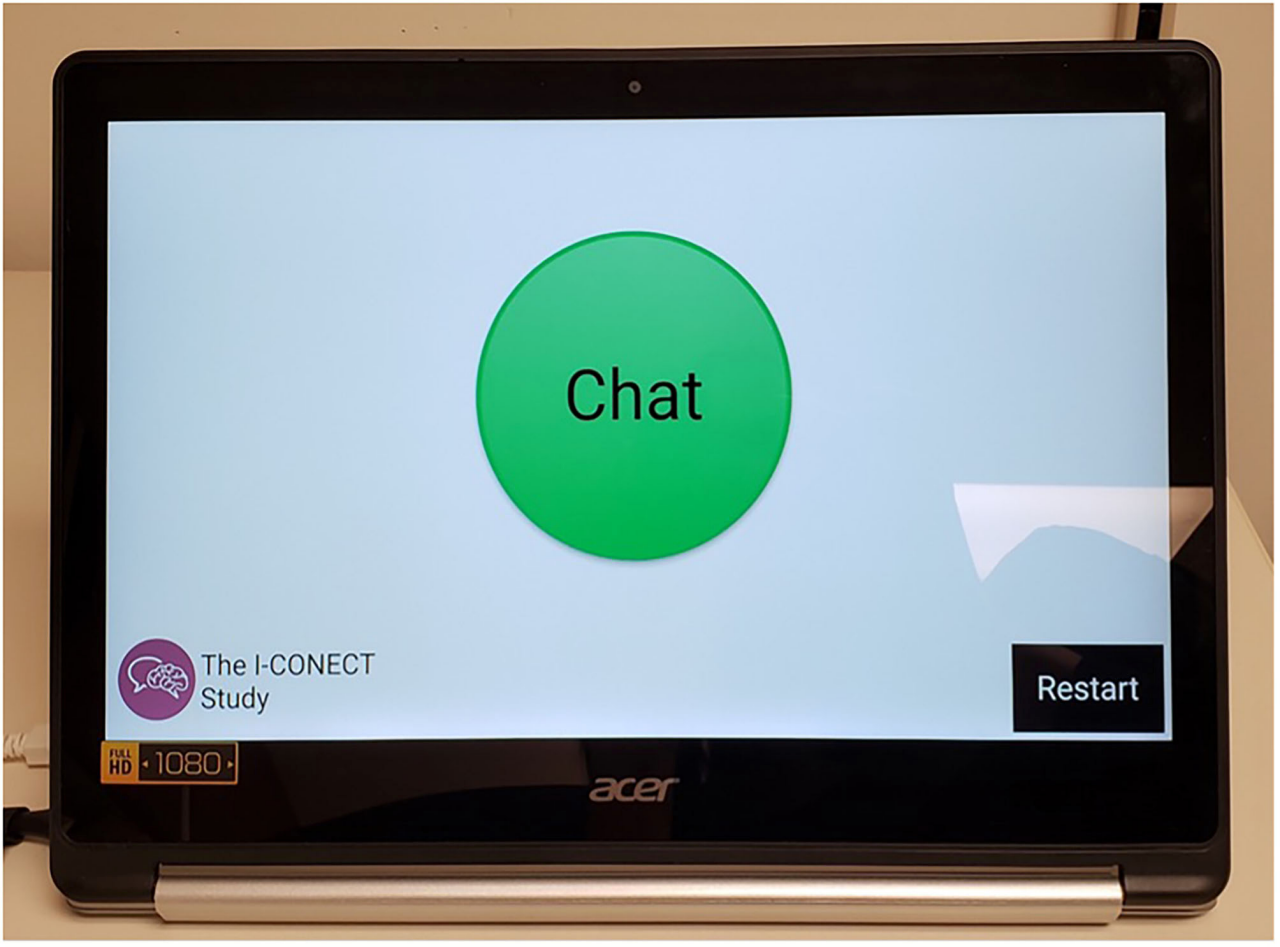
The screen seen by the participant before video chats. We use an entry level 2-in-1 Chromebook with device being managed through Google's G Suite Enterprise for Education for video chats. When calls are coming in, participants touch the green bottom in the middle of the screen to start the video chats. A restart button was placed highly visibly which is important when malfunctions occur and restarting is needed. All video and audio are recorded for analyses with participants' consent (33, 34).

PC usage was a significant predictor of whether subjects were willing to volunteer in our previous study using communication technologies (32). Thus, all potential participants in the sampling pools are reminded that (1) a user-friendly Chromebook will be provided, (2) no previous experience with PC or Internet is required to enroll, and (3) Internet connection will be provided free of charge if they do not have existing Internet service. All equipment necessary for video chats, including Internet service, is provided by the study unless participants already have adequate Internet service and are willing to use their existing Internet service for the study. If this is the case, we reimburse the Internet cost. Figure 2 shows the screenshot of a Chromebook which participants are using for video chats. Participants can touch the green circle to start video chats when the Chromebook rings. Daily picture stimuli are shown during the conversation without any actions required from the participants. When power is unplugged or Internet connection is lost, a warning screen shows up automatically during the chat without interruption. To avoid technical terms like “power adapter” or “audio input” etc., all plugs and ports are color-coded for effective communication with the participants when any technical issues arise.

#### Conversational Materials

The conversation is semi-structured with pre-specified topics with crafted questions to promote natural conversations. Over 150 themes were developed, including topics like historical events, philosophical ideas, social issues, leisure activities and travels. During every session, conversational staff shows three topics under one theme (e.g., under a theme titled “Defining moment of the Twentieth century,” we provide 3 topics participants can select from, including the Great Depression, JFK Assassination, and the Vietnam War). Allowing the participant to select a topic of the day provides him/her a sense of autonomy, which improves motivation to participate in the conversation.

#### Developing Culturally Competent Conversational Materials

In order to recruit African American participants, all the study flyers and advertisement as well as conversational picture stimuli were modified from those used for the white participants to reflect their cultural tradition and heritages. It is well-known that the recruitment of minority groups requires trust and established relationship for successful recruitment. We provided a number of lectures discussing brain health at local community events and circulated study flyers in advance before starting official recruitment.

### Outcomes

A thorough cognitive assessment with standard neuropsychological tests is administered at baseline, 6 months (end point of high dose) and 12 months (end point of maintenance dose) visits, using the National Alzheimer's Coordinating Center (NACC) (https://naccdata.org/) Uniform Data Set version 3 (UDS V3) (35, 36). We also administer the NIH Toolbox (http://www.healthmeasures.net/explore-measurement-systems/nih-toolbox) for within-project cross validation. Both NACC UDS V3 and NIH-Toolbox are developed to assess changes in cognition associated with normal aging as well as ADRD (Alzheimer's Disease and Related Dementias). NIH Toolbox also includes a comprehensive emotion battery that assesses psychological well-being, which will be used to test the hypothesized mediation relationships.

#### Primary Outcomes

Table 2 provides a brief summary of outcomes and hypotheses and Table 3 lists assessment time points for each variable to be collected. Our primary outcome is the change in global cognitive function measured by the Montreal Cognitive Assessment (MoCA) from NACC UDS V3 (23, 37).

**Table 2:** Summary of the outcomes, the assessment tools and specific hypotheses.

	**Assessment tools**	**Hypotheses**
**Primary outcomes**		
Global cognition	MoCA montreal cognitive assessment (MoCA) (Included in the NACC UDS V3)	Within each cognitive group (normal and MCI), the experiment group will experience less cognitive decline or even improvement in global cognitive function compared to the control group
**Secondary outcomes**		
Episodic memory function	Craft Story immediate and delayed recall (included in the NACC UDS V3)	Within each cognitive group (normal and MCI), the experiment group will experience less decline or even improvement in memory functions compared to the control group
Language-based executive function measured	Category Fluency (animals) (CAT) (included in the NACC UDS V3)	Within each cognitive group (normal and MCI), the experiment group will experience less decline or even improvement in executive functions compared to the control group
Psychological well-being	NIH toolbox emotion battery	The intervention group will have more improvements in their psychological well-being compared to the control group. The protential efficacy on cognitive functions through this intervention is partly mediated by improved psychological well-being
Daily functioning	Revised observed tasks of daily living (OTDL-R)	The intervention group will show less decline or even improvement in OTDL-R compared to the control group
**Exploratory outcomes**		
Volume of key regions of interest (ROI)	MRI	Less atrophy or possible increase in volume over time in ROIs will be observed in participants in the intervention group than the control group
Functional connectivity	fMRI	There will be an increase in dorsal attention network (DAN) and Default Model Network (DMN) connectivities among the experimental group in comparison with the control group
Speech utterances and characteristics	Use automated speech algorithms (ASR) to analyze the recorded conversations	Speech characteristics of MCI participants in the experimental group will resemble those with normal cognition over time, compared with the MCI control group participants
Medication adherence	Electronic pillbox that stamps time when the medication is taken	Compared with the control group, medication adherence will increase among the intervention group

**Table 3:** Assessment schedule.

	**Screening period**		**Baseline period**			**Intervention period (weeks 1–48)**		**Follow up period**		
**Time point name**	**Telephone pre-screening**	**Screening**	**Baseline**	**Baseline MRI**	**Tech install**	**Month 6**	**Month 6 MRI**	**Month 12**	**Tech uninstall**	**Telephone follow up**
**Approximate time required**	**30 min**.	**4 h**	**3 h**	**2 h**	**1–2 h**	**4 h**	**2 h**	**4 h**	**30 min**.	**30 min**.
**Scheduling Timeframe**		**10-week window**				**Week 25–26**	**Week 25–26**	**Week 49–50**	**Week 49–50**	**Week 52**
Collect informed consent		X								
Pre-visit stability screening^v^		X	X			X		X		
Collect PCP/contact information^b^		X								
Demographics (NACC form A1)		X								
Social isolation assessment	X	X				X^x^		X^x^		X
GDS (NACC form B6)^t^		X				X		X		X
Neuropsychological battery (NACC form C2)^u^		X				X		X		
CDR (NACC form B4)		X				X		X		
Subject health history (NACC form A5)		X								
Medication assessment		X				X		X		
MRI safety/eligibility screening^a^		X		X			X			
Subject compensation		X^c, w^		X		X^d^	X	X^d^		
Collect NEO-FFI^e^		→	X^e^			X^f^		X^f^		
Clinician diagnosis^s^		X				X		X		
Trial eligibility assessment^h^	X	X								
Post-study resources		X^g^						X		X
Randomization		X^i^							
NIH toolbox			X			X		X		
OTDL-R			X			X		X		
Family history of dementia			X							
Physical evaluation (NACC form B1)			X			X^j^		X^j^		
Saliva collection for APOE ε4			X°							
MRI^q^				X			X			
Vitamin C pillbox^l^					X				X	
Video chat device^m^					X				X	
PANAS^k, m^						X			
Video chat intervention^m^						X (4 times per week for 6 months, 2 times per week for additional 6 months)			
I-CONECT weekly questionnaire^n^						X			
Medication adherence (vitamin C)^l^						X^p^			
Qualitative evaluation (subject)^m^						X**^r^**		X**^r^**		

*NACC, National Alzheimer's Coordinating Center; X, Collected.*

a *At participating sites, subject medical records may be requested if more information is needed to make MRI eligibility determination; final determination may occur any time prior to Baseline MRI*.

b *Subjects may choose to provide emergency contact and primary care provider contact information; this is optional*.

c *Compensation will be provided to all subjects who consent to participate, including screen failures*.

d *Compensation to be provided once all procedures are completed*.

e *The NEO-FFI will be left for subjects to complete independently at the end of the last Screening visit. The questionnaire will be collected and reviewed at the Baseline visit. If the subject did not successfully complete the questionnaire on his or her own, the questionnaire will be completed during the Baseline visit*.

f *If multiple visits will be done to complete all assessments, the questionnaire may be provided at one visit and collected at the subsequent visit. If only one visit is planned for all assessments, the questionnaire may be provided ahead of the visit for collection at the visit*.

g *Resources to be provided at this time point only if subject is a screen failure*.

h *Final eligibility assessment to be completed upon clinician diagnosis*.

i *Randomization to intervention/control occurs once eligibility is determined, prior to the Baseline visit. Subjects should not be notified of group assignment until after Baseline to prevent them from unblinding the assessment staff*.

j *Only blood pressure will be collected at Month 6 and Month 12*.

k *The PANAS will be administered at the beginning and the end of the subject's first video chat session each week*.

l *OHSU subjects only*.

m *Intervention group only. Control group does not receive*.

n *Administered weekly via telephone*.

o *This procedure is optional for subjects. If sample cannot be obtained, collection will be re-attempted at a future visit*.

p *Occurs daily. If the subject misses the target time but remembers the same day, the subject should take the vitamin as soon as they remember. If the subject does not remember until the next day or later, the subject should skip the missed dose and leave the vitamin in the pillbox*.

q *To be completed for subjects who have been assigned to receive MRIs, at participating sites only*.

r *To be completed at the conclusion of the Week 26 and Week 48 video chats. Administration and responses will be audio and video recorded*.

s *The clinician diagnosis must be completed within 1 week once the time point assessments have been completed. Subjects who are diagnosed with dementia must be withdrawn from the trial immediately*.

t *If subject scores 2 or more in response to questions 3, 7, 11,12, or 14 (the suicide ideation subscale), the Depression Safety Assessment will be conducted*.

u *Administration will be audio recorded. Battery assessments must be completed in the order presented in the administration packet to ensure appropriate timing for memory assessments*.

v *If time point assessments are split into multiple visits, this form should be administered at each in-person visit*.

w *Subjects will not receive additional compensation for rescreening*.

#### Secondary Outcomes

Secondary outcomes include language-based executive function measured by Category Fluency (animals) (CAT) (38) and episodic memory function measured by a composite of Craft Story immediate and delayed recall scores (39) from the NACC UDS V3. In our previous pilot project (15), high efficacy was shown in CAT. CAT is generally regarded as a test to tap the executive domain, but it also taps semantic fluency, indicating the integrity of semantic memory (40) and was found to be associated with an amyloid-related decline during the preclinical stage AD (41).

Other secondary outcomes: Other secondary outcomes include changes in (1) psychological well-being measured by the NIH Toolbox emotion battery, (2) daily functioning (translational effects of our intervention on function required in daily living) measured by the Revised Observed Tasks of Daily Living (OTDL-R) (42), and (3) MRI regions of interests (ROI) and fMRI region specific connectivity. Details of the secondary outcomes measures are described below.

##### Psychological Well-Being

The NIH toolbox Emotion battery is used to assess changes in psychological well-being. It includes four general domains: negative affect (anger, fear, and sadness), psychological well-being (positive affect, general life satisfaction, and meaning and purpose), stress and self-efficacy (perceived stress and self-efficacy), and social relationships (social support, companionship, and social distress). The total scores are used to assess changes. We hypothesize that the efficacy shown on cognitive functions through this intervention is partly mediated by improved psychological well-being.

##### Daily Functioning (OTDL-R)

Revised Observed Tasks of Daily Living (OTDL-R) is a performance-based test of everyday problem solving (42). The measurement was used in the Advanced Cognitive Training for Independent and Vital Elderly (ACTIVE) trial to examine the translational effect of intervention to daily living and it is well-validated (43). We hypothesize the intervention group participants would show less decline or even improvement in OTDL-R compared to the control group.

#### Exploratory Outcomes

##### MRI/fMRI

Studies have shown cross-sectional associations between social network size and brain structure (3, 44). To examine underlying mechanisms of our trial efficacy, we examine pre- and post- trial changes in brain structure, function, and perfusion, as exploratory outcomes. Using structural MRI, we characterize the volume of key regions of interest (ROI), specifically hippocampus (HP), amygdala (AG), and cortical thickness of dorsolateral prefrontal cortex (DLPFC). Additionally, to examine potential structural effects beyond these regions, we perform an unbiased whole brain assessment using voxel-based morphometry (VBM). We hypothesize that we will see less atrophy or possible increase in volume over time in participants in the intervention group than in the control group.

Examining functional connectivity using resting-state fMRI, we specifically focus on large-scale brain networks, which support a broad array of cognitive processes including social function (44). We hypothesize an increase in dorsal attention network (DAN) connectivity among the experimental group in comparison with the control group because our intervention involves externally directed attentional tasks using video chat, i.e., which include visual motion area, frontal eye fields, superior parietal lobule, intraparietal sulcus, and ventral premotor cortex. Further, we are interested in connectivity within the default mode network (DMN), which is altered in individuals across the Alzheimer's Disease spectrum (45). DMN is implicated in mentalizing and theory of mind (46), which are critical aspects of effective social interaction. We hypothesize that, in response to the intervention, the experimental group will demonstrate improved or sustained DMN connectivity in comparison to the participants in the control group.

Additionally, we measure brain perfusion using arterial spin labeling (ASL). Blood perfusion is critical for delivery of oxygen and glucose throughout the brain. Reduced cerebrovascular health, common in aging, can affect perfusion and have downstream effects on cognitive performance (47). Therefore, we are interested in assessing brain perfusion as a potential mechanism for trial efficacy under an exploratory analysis.

Other exploratory outcomes include changes in speech utterances and characteristics (acoustic and linguistic features) and medication adherence measured by an electronic pillbox that stamps time when the medication is taken (48). All participants are asked to take one vitamin C tablet per day, at specific time of their choice. We expect that, compared to the control group, medication adherence will increase among the intervention group. Although our team has shown that speech characteristics can distinguish MCI from normal, it has not been used as an outcome in clinical trials (49–51). Since we need to process a large number of recorded conversations, we use automated speech algorithms (ASR) refined in this project to process speech by older old participants in the proposed study. We hypothesize that the speech characteristics of MCI participants in the experimental group will resemble those with normal cognition over time, compared with the MCI in control group participants.

### Development in Personalized Intervention

Humans are heterogeneous with different preferences, and we do not assume one intervention can work for all. It is also important that subjects can sustain the intervention effects after the RCT is over and to maintain the behavior changes imposed during the trials. Therefore, it is of considerable importance to identify characteristics of subjects who can adhere to our intervention and benefit from it. For example, in our previous pilot project, we found different levels of efficacy depend on aspects of participants' personality (17). Additionally, clinical trials efficacy can be dependent on the participants' pathological stages at baseline (52, 53). For example, once disease related atrophy is present, even though participants are clinically determined as pre-symptomatic or early phase of MCI, it might be harder to retard disease progression through behavioral interventions alone. Therefore, in addition to pre-post intervention changes in MRI and fMRI results, baseline MRI and fMRI data are also used to identify phenotypes of “gainers.” Gainers are operationally defined as those who gain in the primary (global cognition) or secondary outcomes (memory and executive domains) at or above 10% in the distribution of gains among the experimental group by applying the regression models derived from the control group to the experimental participants. The model includes age, sex, education, and baseline cognitive test scores.

The following variables will be considered for identifying the characteristics of those who gained: APOE ε4 genotype (from saliva collection), personality (NEO-5 factor inventory) (17), Geriatric Depression Scale (GDS-15 item version) (20), Clinical Dementia Rating (CDR) (54), Grip Strength (measured in NIH toolbox), Subject Health History, medications, MCI sub-types (amnestic MCI vs. non-amnestic MCI, based on clinical diagnosis through neurologist/neuropsychologists consensus meetings), basic physiological assessment (BMI, blood pressure), and MRI ROIs. In exploratory analyses, we will also use machine learning methods to identify features which best identify the gainers, using the methods used in our previous study (55).

### Statistical Approaches

Our protocol has two stages: one under the high-dose period and another under the maintenance dose period. During the maintenance dose period, we will examine whether maintenance dose (30 min conversation twice per week for 6 months) will lead to further improvement in primary and secondary outcomes among the intervention group or at least sustain the improvement observed during the high dose period. Since the maintenance dose requires only half the conversational frequency, if this dose is proven to be sufficient to maintain the gained effect attained during the high does period, it serves as a cost-effective maintenance dose.

We will conduct modified intention to treat (ITT) analysis where those with at least one follow-up assessment under each dose are included in the analysis. We will use changes in primary and secondary outcomes for two separate hypotheses, one for high dose (from baseline to 6 months assessment) and another for low dose (from 6 months assessment to 12 months) using linear regression models where dependent variables are outcomes specified above at month 6 (M6) (controlling for the outcome at baseline) and at month 12 (M12) (controlling for the outcome at M6). For non-continuous outcome variables (e.g., occurrences of missed medications assessed using electronic medication box), we will use generalized linear mixed effects model to estimate the occurrence of events over time. Per protocol analysis (PPT) will be conducted by using the participants who completed at least 80% of the expected sessions under each stage.

### Sample Size Estimates

Our previous pilot study (based on a 6-week intervention) yielded a Cohen's *d* = 0.53 when comparing pre-post changes in language-based executive function measured by the Category Fluency test between the control and experimental groups among participants with CDR = 0 and CDR = 0.5 combined. CDR = 0 group showed even stronger efficacy (Cohen's *d* = 0.68) (15) (ClinicalTrials.gov: NCT01571427). In the previous pilot study, the Mini Mental State Exam (MMSE) was used as an indicator of global cognitive function. In the current study, we use the MoCA for the primary outcome, which has been shown to be more sensitive to subtle cognitive decline associated with MCI than the MMSE (56). Therefore, we expect to achieve at least Cohen's *d* of 0.5 in the current trial in the primary outcome and even larger effect size for the language-based executive function (one of secondary outcomes) based on the pilot study result. We aim to randomize 160 subjects with MCI and 160 subjects with normal cognition (targeted 160 Caucasian and 160 African American participants). Given 20% drop out (10% drop out each between baseline and M6 and between M6 and M12), we expect 128 completers each for those with MCI and normal cognition. With this sample size (*N* = 128, 64 in the control group and 64 in the experimental group), we would have 80% power to detect Cohen's d of at least 0.5 at alpha = 0.05 (two-tailed test) within each diagnostic group.

### Post-COVID Protocol Modification

The COVID-19 pandemic hit the United States in March of 2020. The recruitment sites of this project, Portland, Oregon and Detroit, Michigan issued the first stay-at-home order on March 18 and 23, 2020, respectively. Health care institutions were required to halt any unnecessary medical procedures immediately, which included any in-person contact with research participants. As with other clinical trials, we were unable to enroll new participants through in-person assessments until further notice. Our interventions which use Internet and video chats/phone check-ins have been able to remain as before during this pandemic. However, new recruitment as well as post trial assessments, including MRI, were halted completely since March 23, 2020. Due to the potential high risk of COVID-19 infection among our participants (aged 75 and plus), we were required to process several protocol modifications.

First, we switched our assessment protocol from in-person assessments to complete remote assessments in late June (~3 months after the stay-at-home order). This includes sanitizing all the equipment before delivering to participants, developing color coded easy-to-follow instructions for setting up the equipment, and dropping them off at participants' homes (contactless delivery at the door of the home) and observing from distance to ensure equipment is successfully collected by the participant. Along with changes in logistics of delivering and collecting video chat equipment, we switched to telephone cognitive testing (T-COG, see https://naccdata.org/ for details) upon adaptation the T-COG by the National Alzheimer's Coordinating Center as an alternative to the Uniform Data set version 3 (UDS V3). The T-COG includes the blind MoCA which eliminates visual items from the original MoCA test (https://www.mocatest.org/paper-tests/moca-test-blind/). The modality change is expected to affect cognitive test scores and therefore it is not feasible to examine pre-post changes in certain cognitive tests including MoCA (our primary outcome) and Trail Making Test B (one of secondary outcomes tapping executive functions). Additionally, before switching to remote assessments, participants approaching either M6 or M12 were offered the option to continue in the study at their current level of participation, rather than decreasing chat frequency to maintenance dose or ending chats and/or calls. This extension allowed us to collect assessment data within a reasonable time frame of a subject's final intervention dose, once assessments were able to resume. Although individual participation duration was carefully monitored and will be adjusted for in the statistical analysis, these two major modifications in the study protocol (cognitive assessment modality changes and longer intervention period for some participants whose assessment time were affected by COVID-19 mandatory social distancing order) dictated that we limit our main analysis of this trial to those who completed the end point assessments before March 18, 2020 so that we could: (1) use the outcomes collected with the same modality between baseline and follow-up assessments and (2) avoid contaminated effects of COVID-19 on their emotional status, such as amplified efficacy among the experimental group under the COVID-19-induced social isolation experienced by many older adults in the USA. This decision was suggested by the research team and approved by the DSMB members in September of 2020.

#### Supplemental Analysis

Those who are newly enrolled after March 18, 2020 or those whose 6- or 12-month assessments are conducted remotely due to COVID-19 pandemic will be used for supplemental analyses. In this supplemental phase of the RCT, items omitted from the blind MoCA (and total MoCA scores) will be imputed with the best approach available and used for assessing pre-post changes in the primary outcome. Supplemental analyses will be conducted after main analyses which use only those who completed the assessments before March 18, 2020.

Additional protocol changes include as follows: (1) MRI assessments will be discontinued until COVID-19 related mandatory social distance is lifted. As of 1/1/2021, no MRI assessments have been conducted. (2) the NIH toolbox cognitive battery and grip strength and OTDL-R are no longer administered after COVID-19 due to challenges in standardizing administration. However, the NIH Toolbox Emotional battery is still being collected by delivering a hard copy and asking participants to mail the survey back using pre-stamped envelopes. Follow-up calls are being made to avoid prolonged delay in administration of the survey if participants are late in returning the survey. (3) Due to the cost and lapsed time incurred to maintain staff during the COVID-19 pandemic when no new recruitment and endpoint assessments could occur, it was determined not feasible to continue interventions beyond M6. Although the original proposal aimed to provide reduced dose of intervention (twice per week) for additional 6 months to examine the efficacy of maintenance dose, the study team and DSMB determined that we need to end the trial at 6 months to provide sufficient time to perform data clean up and analyses. This arrangement is also preferred because of assessment modality changes to be faced by the majority of our participants between baseline and M12 assessments. (4) Subjects are required to provide their own, preexisting Internet connection in order to participate in the trial after March 18, 2020, as we cannot safely have Internet vendors enter the homes to install new connections. An Internet stipend is provided.

#### Changes in Targeted Sample Size Due to COVID 19 Protocol Modifications

As discussed above, our main analyses utilize participants who completed M6 assessments before March 18, 2020. Fifty five participants completed both baseline and 6 months assessments before COVID-19 pandemic and these participants will be used for the main analysis. Among the 55 participants, 46 self-identified as non-Hispanic White and 9 identified as non-Hispanic Black. Twenty four participants had normal cognition and 31 were diagnosed with MCI. This sample size is powered to detect Cohen's *d* of 0.8 with 80% power, alpha = 0.05 (two tailed). In the pilot R01 trial (ClinicalTrials.gov: NCT01571427), we found Cohen's *d* of 0.53 after 6 weeks of daily video chats. In our current experiment, the experimental group will experience 6 months of video chats. Therefore, the effect size of 0.8 is not necessarily an overestimation of our trial effect.

#### Timeline of Assessments

We plan to unblind the data for the participants who completed the intervention before March 18, 2020 upon acceptance of this protocol paper. The remaining data will be blinded until we complete all the M6 assessments for all participants. The reports for the main analyses based on the pre-COVID participants and supplemental analyses based on the post-COVID participants are expected to be available by the end of 2021 and 2022, respectively.

## Discussion

Based upon a successful pilot project completed in 2014 (15), the current on-going project provides up to 1 year of conversational interactions via a user-friendly video chat platform, although the COVID-19 pandemic reduced the expected number of participants with 1 year follow-up. Since this is a clinical trial, standardization of conversations is required and therefore we are using trained conversational staff to standardize the talks with participants through video chats. If proven to be effective, we could extend the protocol using peers (instead of trained conversational staff) which could provide cost-effective alternatives. Some of the innovations of our trial are as follows. First, we provide user-friendly hardware so that the participants who do not have any experience in using a PC or laptop can participate. Second, conducting interventions in their homes increases adherence and sustainability even among older old participants who often have limitations in mobility. As an example, our RCT could continuously provide needed social interactions among the existing participants during the recent COVID-19 pandemic. Third, most interventions thus far target younger old volunteers (aged 65–75) while older old or those with social isolation are rarely involved in clinical trials. Considering the negative effects of social isolation on health and age as one of the strongest risk factors of cognitive decline, targeting this underserved group is of high public health importance. Ultimately, we hope to use our conversational materials and related platform as a treatment for social isolation and resultant cognitive decline. Fourth, we examine translational effects focusing on medication management, one of the most important IADL items for living independently in the community, which declines at an early stage of cognitive impairment. We assess this ability using a validated objective measure (OTDL-R) as well as an electronic pillbox, one of the digital biomarkers which provided the association with cognitive functions. Fifth, exploratory analyses on changes in speech characteristics could yield promising behavioral markers of early cognitive decline, responding to the urgent need to identify ecologically valid outcomes sensitive to trials effects in the dementia research field (3). Finally, while exercise-based interventions often aim to examine the underlying biological mechanisms of efficacy (57), how and why social interaction affects cognition is yet to be discovered. We will use baseline characteristics of brain structures and connectivity to investigate who gains most in addition to the intervention efficacy (pre-post changes).

There are some limitations in the study design. It would be ideal to have a larger sample size for each racial group since there might be cultural differences that differentiate the levels of efficacy. Even though we aim to recruit 50% African American participants, once we stratify the analysis by cognitive status (MCI vs. normal), the sample size within each strata diminishes quickly. Duration of follow-up is also limited up to 1 year (and modified as up to 6 months after COVID-19 pandemic) even though most epidemiological studies that provided evidence in the past for the association between social engagement and cognition had been following participants from mid-life to late-life. That said, this RCT can test whether enhancing social interactions through video chat can improve cognitive function at later life and open a new venue for cost-effective home-based behavioral interventions in the community.

## Ethics Statement

The studies involving human participants were reviewed and approved by Institutional Review Board at the Oregon Health & Science University. The patients/participants provided their written informed consent to participate in this study.

## Author Contributions

HD, KW, KP, BH JK, LMS, PP, PL, EA, JL, MM, and LCS contributed to the conception and design of the study. KY and HD contributed to draft and edit the full manuscript. HD received the funding. All authors contributed to manuscript revision, read, and approved the submitted version.

## Conflict of Interest

MM was employed by company Syneos Health. The remaining authors declare that the research was conducted in the absence of any commercial or financial relationships that could be construed as a potential conflict of interest.

## Publisher's Note

All claims expressed in this article are solely those of the authors and do not necessarily represent those of their affiliated organizations, or those of the publisher, the editors and the reviewers. Any product that may be evaluated in this article, or claim that may be made by its manufacturer, is not guaranteed or endorsed by the publisher.
