# Supplementary material for: The Internet-Based Conversational Engagement Clinical Trial (I-CONECT) in Socially Isolated Adults 75+ Years Old: Randomized Controlled Trial Protocol and COVID-19 Related Study Modifications
Source: Front Digit Health. 2021 Aug 25;3:714813. doi: 10.3389/fdgth.2021.714813 (PMC8521795; doi:10.3389/fdgth.2021.714813)
Supplement: Supplementary file 1 [file Table_1.docx]

Appendix 1. Operational Criteria Used to Define “Being Social Isolated or Lonely” (I-CONECT project: <https://www.i-conect.org/>)

#1. LONELINESS ASSESSMENT

A three-item version of the UCLA Loneliness Scale developed for use in large-scale epidemiological studies

Responses: 1. Hardly Ever 2. Some of the Time 3. Often

Questions:

1. How often do you feel that you lack companionship: Hardly ever, some of the time, or often? 1 2 3

2. How often do you feel left out: Hardly ever, some of the time, or often?

1 2 3

3. How often do you feel isolated from others? (Is it hardly ever, some of the time, or often?) 1 2 3

Include the subject if at least one item was scored as “often”.

Hughes, M. E., Waite, L. J., Hawkley, L. C., & Cacioppo, J. T. (2004). A Short Scale for Measuring Loneliness in Large Surveys: Results From Two Population-Based Studies. Research on Aging, 26(6), 655–672. <https://doi.org/10.1177/0164027504268574>

**OR**

#2 LUBBEN SOCIAL NETWORK SCALE – 6 (LSNS-6)

I would like to ask you about your frequency of social interactions. I have 6 brief questions.

FAMILY: Considering the people to whom you are related by birth, marriage, adoption, etc…

1. How many relatives do you see or hear from at least once a month?

0 = none 1 = one 2 = two 3 = three or four 4 = five thru eight 5 = nine or more

2. How many relatives do you feel at ease with that you can talk about private matters?

0 = none 1 = one 2 = two 3 = three or four 4 = five thru eight 5 = nine or more

3. How many relatives do you feel close to such that you could call on them for help?

0 = none 1 = one 2 = two 3 = three or four 4 = five thru eight 5 = nine or more

FRIENDSHIPS: Considering all of your friends, including those you live in your neighborhood…

4. How many of your friends do you see or hear from at least once a month?

0 = none 1 = one 2 = two 3 = three or four 4 = five thru eight 5 = nine or more

5. How many friends do you feel at ease with that you can talk about private matters?

0 = none 1 = one 2 = two 3 = three or four 4 = five thru eight 5 = nine or more

6. How many friends do you feel close to such that you could call on them for help?

0 = none 1 = one 2 = two 3 = three or four 4 = five thru eight 5 = nine or more

Total score ≤ 12 (based on the thresholds defined by Lubben et al., )

J. Lubben, E. Blozik, G. Gillmann, S. Iliffe, W. R. Von Kruse, J. C. Beck and A. E. Stuck, Gerontologist 2006, 46, 503-513.

**OR**

# 3 FREQUENCY OF CONVERSATION

“In a typical week, how often do you have a conversation that lasts for 30 minutes or longer?”

1. Almost never

2. Once or twice

3. Three to five times

4. Almost daily

Need to score 1 or 2
